# Effects of Print Exposure on an Online Lexical Decision Task: A Direct Replication Using a Web-Based Experimental Procedure

**DOI:** 10.3389/fpsyg.2021.710663

**Published:** 2021-08-11

**Authors:** Dahyeon Kim, Matthew W. Lowder, Wonil Choi

**Affiliations:** ^1^Department of Life Sciences, Gwangju Institute of Science and Technology, Gwangju, South Korea; ^2^Department of Psychology, University of Richmond, Richmond, VA, United States; ^3^Division of Liberal Arts and Sciences, Gwangju Institute of Science and Technology, Gwangju, South Korea

**Keywords:** web-based experiment, Korean author recognition test, print exposure, individual differences, lexical decision

## Abstract

Due to the global pandemic, behavioral sciences including psychology that have traditionally relied on face-to-face data collection methods are facing a crisis. Given these circumstances, the present study was designed as a web-based replication of the findings reported in Lee et al. (2019) on the relationship between print exposure measured by the Korean Author Recognition Test (KART) and online measures of word processing using the lexical decision task and offline measures of language ability. We used the PsychoPy3 and Pavlovia platform in which participants were presented with a series of tasks in an entirely web-based environment. We found that scores on the KART were correlated with scores on a measure of language skills as well as self-reported reading habits. In addition, KART scores modulated the word frequency effect in the lexical decision task such that participants with higher KART scores tended to have smaller frequency effects. These results were highly consistent with previous lab-based studies including Lee et al. indicating that web-based experimental procedures are a viable alternative to lab-based face-to-face experiments.

## Introduction

### Author Recognition Test as a Measure of Print Exposure

A large body of research has demonstrated that an individual's degree of exposure to printed language is closely related to a wide variety of language outcome measures. These effects of print exposure exist across the lifespan, beginning as early as preschool. For example, Mol and Bus (2011) conducted a meta-analysis on the association between print exposure and language abilities across three age groups—preschoolers and kindergarteners, children in Grades 1–12, and undergraduate and graduate students, demonstrating robust effects across all groups. In addition, exposure to printed language through home literacy activities in early childhood can help develop language-related skills such as vocabulary size (Mol et al., 2008), as well as phonological and orthographic processing of words (Share, 1999) and vocabulary knowledge (Cunningham and Stanovich, 1991). Beneficial effects of print exposure have also been well-documented among adult readers, showing associations with verbal fluency, vocabulary knowledge, and spelling knowledge (Stanovich and Cunningham, 1992), and also with general knowledge (Stanovich and Cunningham, 1993). Researchers have explained these findings in cyclical terms such that more time spent reading (or being read to at a young age) can increase an individual's language skills, which can then lead to the individual being more likely to accept the reading activity itself as enjoyable, which leads to more time spent reading, which in turn can continue to improve the individual's language skills (West et al., 1993; Mol and Bus, 2011). In this way, print exposure has been noted as both a cause and an effect of language ability, which makes it a particularly interesting variable for research examining individual differences in language ability.

The Author Recognition Test (ART) developed by Stanovich and West (1989) is the most commonly used measure for estimating an individual's degree of print exposure. In the ART, participants are presented with a list consisting of names of authors and foils and are instructed to mark the names they recognize as authors. To correct for guessing, the number of foils selected is subtracted from the number of authors selected. The ART is based on the assumption that people who read frequently have richer knowledge of literature and authors and therefore will recognize more author names than people who read less frequently. The ART has been found to have high reliability (Cronbach α = 0.84 in Stanovich and West, 1989). It also has the advantage of being free from the effects of socially desirable responding, in which participants might misrepresent how much time they spend reading if asked to self-report (Stanovich and West, 1989).

There is a substantial body of literature indicating relationships between ART scores and many language-related abilities, including word-decoding skills associated with reading as well as higher-level skills associated with vocabulary knowledge. For example, higher ART scores are associated with enhanced word-recognition performance (Stanovich and West, 1989), faster self-reported reading time (Acheson et al., 2008), and increased vocabulary knowledge (Stanovich and Cunningham, 1992). Regarding online processing measures, studies employing the lexical decision task have reported that participants with higher ART scores tend to show faster response times, better accuracy, and a smaller word frequency effect compared to participants with lower ART scores (Chateau and Jared, 2000; Sears et al., 2006). This finding regarding differences in the magnitude of the word frequency effect is particularly noteworthy, as it shows that not only do individuals with higher levels of print exposure make faster and more accurate lexical decisions, but they also access lexical information more efficiently. The idea is that as individuals read more, they tend to encounter low-frequency words more often, which in turn reduces the size of the word frequency effect. Finally, studies recording eye-movement behavior during reading have shown that readers with higher ART scores tend to show higher skipping rates, shorter first-pass reading times, and larger perceptual spans as compared to those with lower ART scores (Choi et al., 2015; Moore and Gordon, 2015; Lowder and Gordon, 2017; Gordon et al., 2020).

The findings described above demonstrating that higher levels of print exposure are related to better performance on a wide variety of language-related outcomes are broadly consistent with the *lexical quality hypothesis* (Perfetti, 1985, 2007). According to the lexical quality hypothesis, increases in language exposure beginning from a young age strengthen an individual's orthographic and lexical knowledge. These high-quality lexical-level linguistic representations then form the foundation for higher-level semantic representations that allow for increases in vocabulary knowledge. Given that the ART is considered a reliable and valid estimate of an individual's exposure to printed language (Stanovich and West, 1989), it serves as a useful measure of how much reading practice an individual has had. Under the lexical quality hypothesis, increased levels of reading practice give rise to the high-quality linguistic representations that are believed to strengthen the associations between lower-level skills of orthographic decoding and higher-level knowledge of word meanings.

Because knowledge about authors and literature is culturally specific, it is important that items on the ART be appropriately selected for the population of interest. Accordingly, the ART has been successfully developed in several other languages to be used as a measure of print exposure for non-English speaking populations around the world. For example, Brysbaert et al. (2020) developed a Dutch author recognition test (DART) consisting of 90 real author names and 42 foils. DART scores were found to be associated with several measures of language ability, such as vocabulary knowledge and spelling knowledge, and has been reported as a reliable and valid test among Dutch and Belgian populations. In addition, Chen and Fang (2015) developed the Chinese Author Recognition Test (CART) for use in Taiwan. The CART consists of 75 real author names and 75 foils, and CART scores were found to be associated with vocabulary knowledge, reading comprehension, and Chinese General Scholastic Ability Test scores.

The Korean Author Recognition Test (KART) was recently developed and tested in a lab-based setting by Lee et al. (2019). Consisting of 40 real author names and 40 foils, KART scores were shown to be significantly correlated with self-reported comparative reading habits (CRH) scores (intraclass correlation coefficient = 0.42), and also with language abilities such as vocabulary knowledge (intraclass correlation coefficient = 0.35) and reading comprehension ability (intraclass correlation coefficient = 0.31). Furthermore, higher KART scores were found to be associated with faster lexical decision response times and smaller word frequency effects.

Given the robust relationships that have been reported between print exposure measured by the ART and various language-related variables, it is reasonable to expect that similar associations will emerge using a web-based experimental procedure. Thus, the goal of the current study was to attempt to replicate and extend the results of Lee et al. (2019) using a new sample of Korean participants in an entirely web-based format, as opposed to inviting participants to a laboratory. This goal is particularly relevant given our current situation in which the COVID-19 global pandemic has made conventional laboratory behavioral experiments difficult or impossible to conduct. As more and more behavioral studies are conducted on web-based platforms, it becomes increasingly important to verify that the effects obtained on the web are equivalent to effects obtained in the lab.

### Web-Based Experimental Procedures and Directions of the Current Study

Over the past decade, we have seen a remarkable increase in the popularity of conducting behavioral research via web-based experiments. This approach has the advantage of being able to reach a larger and more diverse population compared to traditional lab-based experiments in a faster and more economical way (Kochari, 2019). At the same time, research conducted using web-based experiments has also been regarded with a high degree of skepticism, with critics arguing that the timing of stimulus presentation or response recording may be inaccurate (Woods et al., 2015).

However, in recent years, with the improvement of the processing power of web browsers and computers, concerns about the timing of web-based experiments may no longer be a serious problem (Reimers and Stewart, 2015). Indeed, web-based experiments still tend to show slower latencies with more variability compared to laboratory-based experiments; however, many studies have provided evidence that the response time data obtained from web-based experiments are sufficiently reliable (Barnhoorn et al., 2015; Reimers and Stewart, 2015; Miller et al., 2018; Anwyl-Irvine et al., 2021). Furthermore, many well-established effects in cognitive science such as Stroop, Flanker, Simon, and visual search have been successfully replicated using web-based experiments (e.g., Barnhoorn et al., 2015; Hilbig, 2016; Semmelmann and Weigelt, 2017), supporting the general idea that data collected from web-based experiments are valid for use in research.

Several tools have also been developed to create and conduct web-based experiments without an advanced understanding of computer science and browser technology. The present study used PsychoPy3 (Peirce et al., 2019), an open-source local application that provides an experiment builder and Python programming library. PsychoPy3 enables researchers to create experiments, export them to Pavlovia.org (a web platform based on GitLab), and collect experimental data. In addition to PsychoPy3, several other tools are available for researchers to create web-based experiments, including Gorilla, Lab.js, and PsyToolKit. The various applications differ in terms of the functions provided, the programming language used, and the depth of required programming knowledge.

Accordingly, the goal of the current study was to determine whether the lab-based results of Lee et al. (2019), which reported that the magnitude of the word frequency effect in lexical decision response times was modulated by KART scores, would replicate using a web-based experimental procedure. Given that the KART, Comparative Reading Habits (CRH), and language skills task are survey-based and thus do not require precise measurement of response times, we predicted that these measures would yield reliable and valid results. In line with this prediction, Gosling et al. (2004) have reported that reliable data can be obtained from web-based surveys and questionnaires. Considering the results of previous studies (Simcox and Fiez, 2014; Hilbig, 2016), it can be expected that reliable and valid results can be obtained through web-based lexical decision tasks as well. For example, Hilbig randomly assigned participants to three lexical decision task conditions: (a) using standard experimental software (E-Prime) in the lab, (b) using a browser-based version in the lab, and (c) using the same browser-based version on the web. Results showed evidence of a word frequency effect across all three conditions. Additional studies have reported that the word frequency effect emerges in crowdsourced, web-based lexical decision megastudies in both Spanish and English, similar to effects that emerge in lab-based experiments (Aguasvivas et al., 2020; Mandera et al., 2020).

In summary, the current study addresses two primary research questions. Our first question was whether individual differences in print exposure assessed via the KART would modulate the magnitude of the word frequency effect, as measured via a lexical decision task. Second, we sought to directly replicate the results of Lee et al. (2019) lab-based experiment using entirely web-based experimental procedures.

## Materials and Methods

### Subjects

One hundred fifty-three undergraduate and graduate students from two colleges were recruited as participants. Five participants with an accuracy rate on the lexical decision task of <70% were excluded from the analysis. Thus, data from 148 participants were used for the analysis (90 females). Participants who were included in the analysis were 18–28 years old, and the mean age was 20.93 years (*SD*: 1.91). Eighty-eight students (59.46%) were students from Yeungnam University, and the other sixty students (40.54%) were students from Gwangju Institute of Science and Technology. All participants were native speakers of Korean.

### Materials

For the lexical decision task (LDT), the same 120 Korean words and 120 non-words that were used in Lee et al. (2019) were used in the current study. The log-transformed mean written frequency of the word stimuli used in the analysis was 5.3 (*SD*: 1.96). The average length of the word stimuli was 2.06 syllables (*SD*: 0.23). Word frequency information was not available for 12 of the words. Thus, the 108 word stimuli for which word frequency information was available were used for the analyses.

### Individual Difference Tasks

#### Korean Author Recognition Test

The Korean Author Recognition Test (KART) developed by Choi and colleagues (Lee et al., 2019), was used in the current study. In this test, participants are presented with 80 names and are instructed to select the ones they know are authors. Forty of the names are actual authors, and the other 40 are foils. The score is computed by subtracting the number of foils selected from the number of real authors selected. This measure was presented to participants using Google Forms.

#### Comparative Reading Habits

Comparative Reading Habits (CRH) is a self-report questionnaire that asks participants to estimate their own reading habits in comparison with their peers. Each item is presented on a Likert scale ranging from 1 to 7, with higher scores indicating better reading habits than their peers. The CRH consists of five items: amount of time spent reading, complexity of the reading material, reading enjoyment, reading speed, and reading comprehension ability. The English version of the CRH used in Acheson et al. (2008) was translated into Korean and used in the current study. The item asking about amount of time spent reading was edited to ask about the amount of time spent reading when compared to their peers. This measure was presented to participants using Google Forms.

#### Language Skills Task

To evaluate language skills, participants were presented with 40 multiple-choice questions. These items included questions about word meanings and spelling, as well as knowledge about proverbs and idioms. For example, the 28 items measuring word meanings included questions asking to choose the word whose meaning is the furthest from the other three among the four options. In addition, the nine questions about spelling asked to choose the correct spelling among four examples with similar pronunciation. The two questions about proverbs asked to choose the appropriate option that completed the proverb among four options with similar meanings. Finally, the one question about idioms asked to select the appropriate idiom to fill in the blank in a sentence. Scores were calculated as the number of correct answers out of 40. A web-based experimental version of the task was created using PsychoPy3 (Peirce et al., 2019).

#### Procedure

The current study was performed following the experimental procedure of Lee et al. (2019). Lee et al. (2019) the participants visited the lab, and all tasks were administered by an experimenter. In contrast, all procedures in the current study were conducted through a web-based experimental procedure in which participants performed the tasks by themselves in their homes or in a setting of their choosing. The order of each task was the same across the two studies. The experimental procedure of the current study is described in more detail as follows.

Ethical approval for the current study was granted by the Gwangju Institute of Science and Technology IRB. After providing informed consent, the URL to participate in the web-based experiments was sent to the participant via email.

In the LDT, participants were presented with letter strings and were instructed to decide whether or not each letter string was a real word. At the beginning of each trial, a fixation cross (+) was presented in the center of the screen for 1,000 ms. Then, the stimulus was presented until the participant responded. Participants performed 20 practice trials and then responded to the 240 stimuli, which were presented randomly. Participants pressed the “a” key if they thought the stimulus was a real word, and the “l” key if they thought it was not a word. They were instructed to respond as quickly as possible without sacrificing response accuracy.

Following the LDT, participants completed the KART, the CRH questionnaire, and the language skills task. The entire experimental session took ~45 min.

## Results

### Analyses

KART scores were calculated by subtracting the selected number of foils from the selected number of actual authors. CRH scores were calculated as the mean of all five items in the questionnaire. The language skill scores were calculated as the number of correct answers out of 40 questions. For the LDT reaction time data, trials with a response time of <300 ms or more than 2,000 ms were excluded from the analysis. In addition, incorrect trials were excluded from analysis of the reaction time data. Using these criteria, out of a total of 15,984 trials, 14,487 trials were included in the analysis (9.37% of data were excluded). Descriptive statistics^1^ for all measures are presented in Table 1.

**Table 1:** Means, standard deviations, and ranges of each measure.

	**Mean (*SD*)**	**Range**
KART	14.4 (8.1)	0–37
CRH	3.6 (1.2)	1–6.4
LS	21.2 (5.3)	7–36
LDT.RT (milliseconds)	694 (251)	312–1,987
LDT.ACC	0.93 (0.05)	0.76–1.00

*KART, Korean author recognition test score; CRH, comparative reading habit (average value); LS, language skills test score; LDT.RT, lexical decision task reaction time (trimmed); LDT.ACC, lexical decision task accuracy*.

The reaction time data from the LDT were analyzed using a linear-mixed effects model. The lmer/glmer function of the lme4 package (Bates et al., 2015) in R (R Development Core Team, 2021) was used for the analysis. The lmerTest package (Kuznetsova et al., 2017) was used to obtain *p*-values. To analyze the relationship between KART scores and LDT performance, the fixed effects included in the analysis were the written word frequency, KART score, and the interactions among the two factors. Both fixed effects were continuous variables. Subjects and items were included as crossed random effects.

### Correlations Between KART and Other Measures

**Figure 1:**
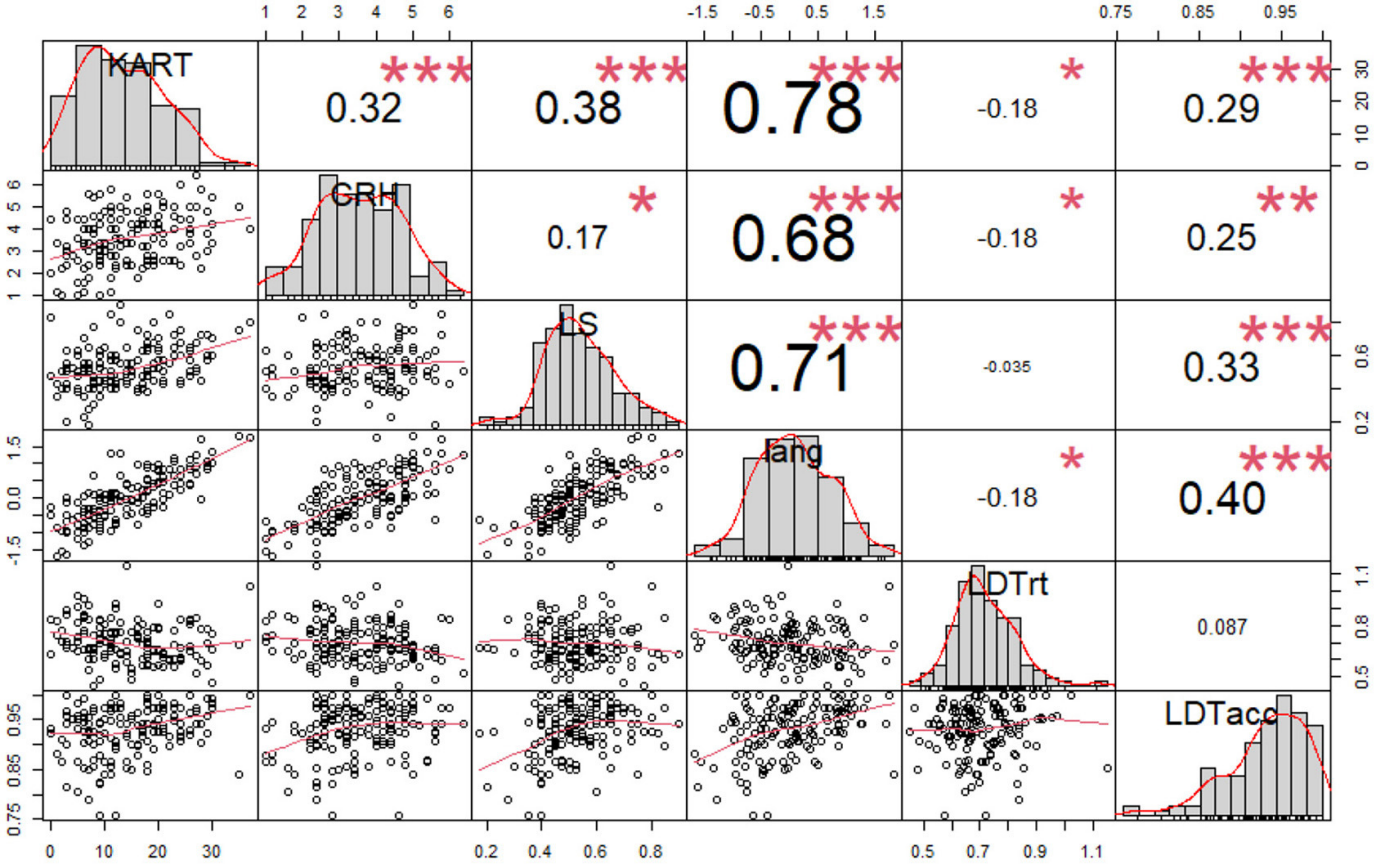
Correlations between KART and other measures. *p < 0.05; **p < 0.01; ***p < 0.001. The diagonal values are histograms of the distribution of each variable. The upper values of the diagonal are correlation coefficients and the lower values are scatter plots. KART, Korean author recognition test score; CRH, comparative reading habit (average value); LS, language skills test score; lang, composite variable of KART, CRH, LS (average *z* score of KART, CRH, LS); LDTrt, lexical decision task reaction time; LDTacc, lexical decision task accuracy.

The results of correlation analyses between KART scores, the other individual difference measures, LDT response time, and LDT accuracy are presented in Figure 1. As illustrated in the figure, KART scores showed a moderate positive correlation with scores on the other individual difference measures. Specifically, the correlation between KART scores and the average CRH scores was 0.32, and the correlation between the KART scores and LS scores was 0.38. The magnitude of the correlation coefficients is similar to the results obtained in the original study (*r* between KART and CRH = 0.41, *r* between KART and LS = 0.35 in Lee et al., 2019).

KART scores also showed a significant correlation with LDT reaction time and accuracy. KART and LDT reaction time were negatively correlated, whereas KART and LDT accuracy were positively correlated, indicating that higher KART scores were associated with faster and more accurate LDT responses. In other words, increased exposure to printed language is associated with better word recognition performance.

### LDT Data Modulated by KART

**Figure 2:**
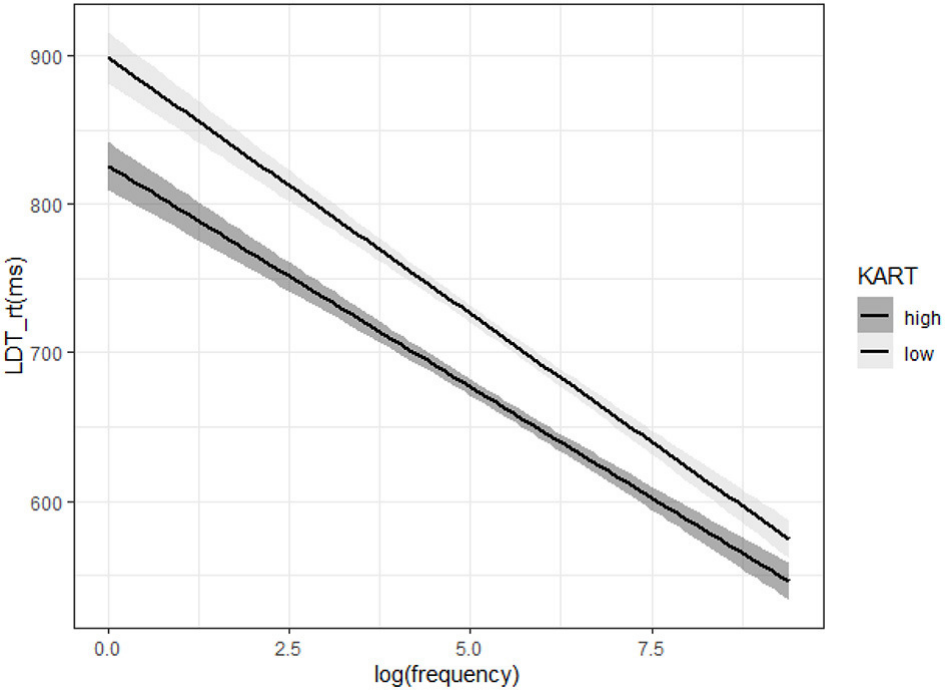
Effects of word frequency and KART scores on reaction time (ms) in the lexical decision task. Note that the KART score variable was entered into the model as a continuous variable in the analysis. This variable is shown as a median split in the figure for the purposes of illustration.

Results of the linear mixed effects model analysis of LDT reaction time data revealed a main effect of KART scores (*ES* = −31.23, *SE* = 10.42, *t* = −3.00, *p* < 0.01), indicating that higher KART scores were associated with faster LDT reaction times. The analysis also revealed a main effect of word frequency (*ES* = −36.60, *SE* = 3.49, *t* = −10.49, *p* < 0.001), indicating that increases in word frequency were associated with faster reaction times. Notably, there was a significant interaction between KART scores and word frequency (*ES* = 2.03, *SE* = 0.87, *t* = 2.139, *p* < 0.05). As can be seen in Figure 2, the effect of word frequency on response times was modulated by KART scores. Participants with higher KART scores tended to show smaller word frequency effects than those with lower KART scores. In other words, participants with lower levels of print exposure took longer to process low-frequency words compared to high-frequency words, whereas participants with greater amounts of print exposure showed a smaller difference in response time between high- and low-frequency words.^2^

**Figure 3:**
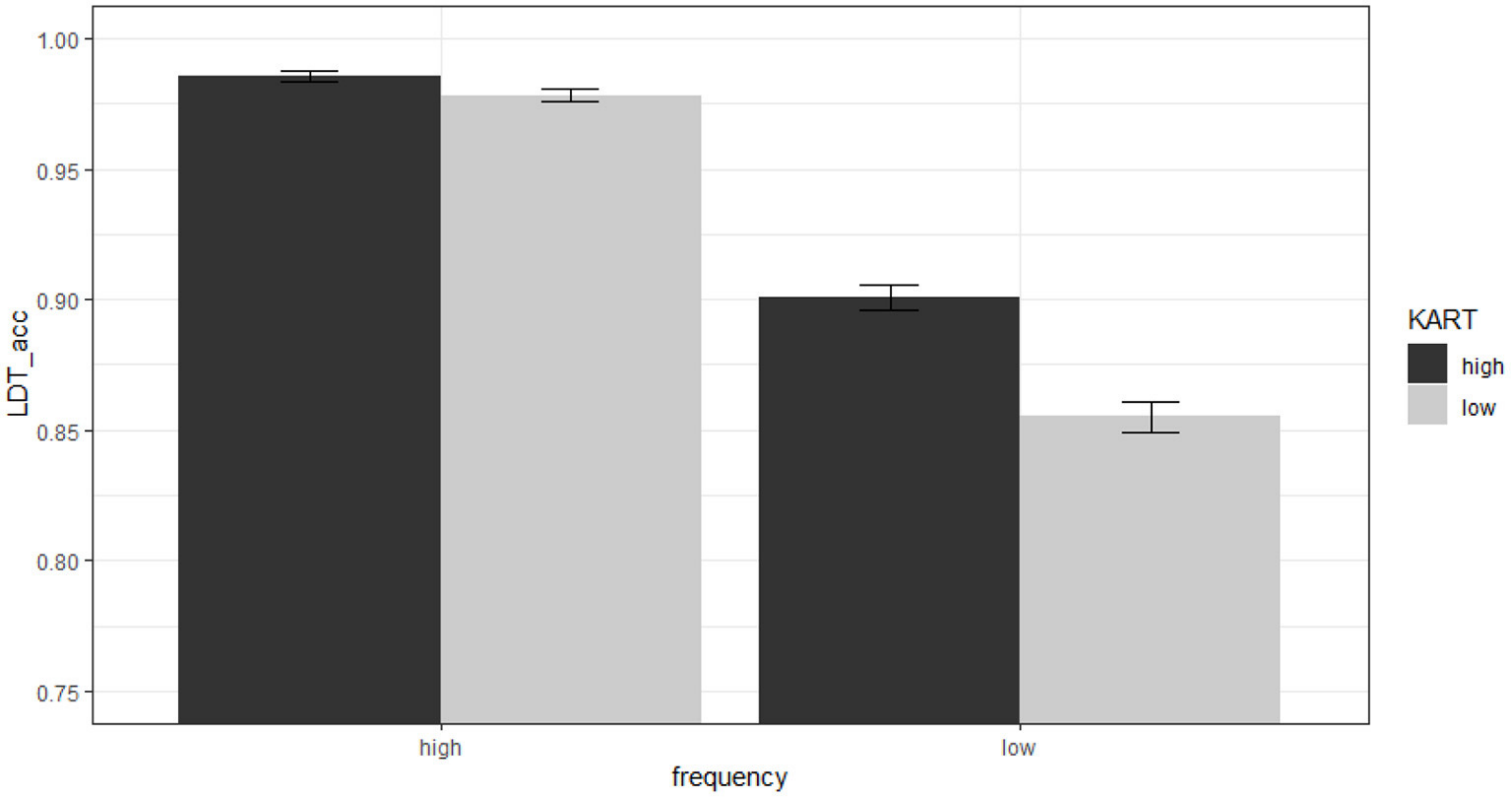
Effects of word frequency and KART scores on accuracy in the lexical decision task. Error bars represent standard error (*SE*) of the mean. Note that word frequency and KART scores were entered into the model as continuous variables in the analyses. They are shown as median splits in the figure for the purposes of illustration.

Results of the LDT accuracy data also revealed a main effect of KART scores (*ES* = 0.50, *SE* = 0.11, *z* = 4.32, *p* < 0.001), indicating that higher KART scores were associated with higher LDT accuracy. The analysis also revealed a main effect of word frequency (*ES* = 0.54, *SE* = 0.066, *z* = 8.23, *p* < 0.001), indicating that higher word frequency was associated with higher LDT accuracy. More importantly, there was a significant interaction between word frequency and KART scores (*ES* = −0.048, *SE* = 0.021, *z* = −2.32, *p* < 0.05). As can be seen in Figure 3, the effect of word frequency on LDT accuracy was modulated by KART scores. Specifically, participants with higher KART scores showed a smaller effect of word frequency on LDT accuracy than those with lower KART scores. In other words, participants with lower levels of print exposure had decreased LDT accuracy for low-frequency words compared to high-frequency words, whereas participants with higher levels of print exposure showed a reduced LDT accuracy difference between high- and low-frequency words.^3^

## Discussion

In the present study, we attempted to directly replicate the findings reported in Lee et al. (2019) showing that an individual's degree of print exposure measured by the Korean Author Recognition Test (KART) was correlated with other measures of language skill, and that KART scores also modulate the magnitude of word frequency effects as measured by a lexical decision task. The key difference between Lee et al. and the present study was the environment in which the experiment was conducted. Whereas, Lee et al. (2019) experiment was conducted in a well-controlled lab environment, the present study was conducted in an entirely web-based environment, which necessitates the loss of a great deal of experimental control. Despite the web-based environment, the results reported here serve as a successful replication of all the key results reported in Lee et al. Our findings provide important evidence that online lexical decision data and offline language skill measures collected via a web-based platform yield valid and reliable results. It is important to note that there is a qualitative difference between the lexical decision task and the language skills task that were used in the current study. Although both tasks are related to lexical retrieval, the lexical decision task reflects the speed of lexical access as well as the accuracy of retrieving stored lexical representations from memory, whereas the language skills task is an indication of how much information an individual has about the meanings of words without time pressures. As seen in Figure 1, the relationship between language skills test scores and LDT accuracy was weak to moderate, whereas the relationship between language skills scores and LDT reaction times was not meaningful. This pattern further suggests that the two tasks are measuring different aspects of language-related abilities.

Table 2 presents correlation coefficients between ART scores and other measures of language ability from the current study as well as several others. As indicated in the table, ART scores show moderate correlations with CRH scores, ranging from 0.30 to 0.41. In addition, ART scores and Vocabulary scores also showed reliable correlations, ranging from 0.23 to 0.42. The current study also reported a reliable correlation between ART scores and LDT accuracy, which is consistent with the results of Lee et al. More importantly, the current study also replicated the interaction between KART scores and the word frequency effect reported in Lee et al. such that people with higher KART scores tended to have a smaller difference in the time required to recognize high-frequency words vs. low-frequency words, compared to people with lower KART scores. Overall, the correlations between KART scores and other measures reported in the current study are consistent with the results of previous studies. Crucially, the results of Lee et al. were replicated in the current web-based experiment, providing evidence that web-based experimental procedures yield reliable data.

**Table 2:** Summary table of correlations between author recognition tests and other measures in recent studies.

		**Current study**	**Lee et al. (2019)**	**Chen and Fang (2015)**	**Brysbaert et al. (2020)**
Language		Korean	Korean	Chinese	Dutch
Experiment type		Web-based	Lab-based	Lab-based	Lab-based
Correlation with other measures	CRH	0.32	0.41	0.30	
	LS	0.38	0.35	0.23	0.42
	LDT.ACC	0.29	0.39	–	–

*CRH, comparative reading habit (average value); LS, language skills test score; LDT.ACC, lexical decision task accuracy*.

In Lee et al. (2019), the mixed effects model analyzing the effects of KART scores and word frequency on LDT accuracy did not converge, so the relationship was verified using a two-factor ANOVA. In the current study, in contrast, the mixed effects model did converge. The most likely reason for this difference in model convergence is the number of participants tested (148 in the current study; 104 in Lee et al., 2019). Regardless, both studies found similar main effects of KART and word frequency, and the interaction between these two factors also showed the same pattern. That is, in the case of high-frequency words, KART scores did not modulate the difference in LDT accuracy, whereas in the case of low-frequency words, the higher the KART score, the higher the LDT accuracy. This pattern is consistent with other studies showing that individuals with higher ART scores tend to have smaller word-frequency effects compared to individuals with lower ART scores (Chateau and Jared, 2000; Sears et al., 2006), indicating that individuals with higher levels of print exposure access lexical information more efficiently than those with lower levels.

We also found that the overall reaction time tended to be slightly delayed in the web-based experiment. Mean reaction time of the raw data in the lab-based LDT experiment reported by Lee et al. (2019) was 705 ms, whereas mean reaction time of the raw data in the current web-based LDT experiment was 804 ms, showing a delay of about 100 ms. Several factors may have contributed to this difference such as participants becoming distracted in an unsupervised environment as well as inaccuracies in the response time recording, which are problems that have been continuously raised with web-based experiments. In particular, it has been reported that the response times recorded by keyboards have a delay of about 11 to 73 ms compared to response devices that are generally used in lab experiments (Shimizu, 2002). Also, in the web-based experiment environment, each participant uses different systems including CPU, RAM, monitors, and keyboards. Considering that different keyboards can have different delay times, it is reasonable to expect that the variance in response times will also be larger in web-based experiments (Kochari, 2019). In fact, this temporal delay is commonly found in web-based experiments (Reimers and Stewart, 2015; de Leeuw and Motz, 2016; Hilbig, 2016; Semmelmann and Weigelt, 2017). In a study that compared the lab-based LDT with the browser-based LDT, the average response time of the lab-based experiment was 961 ms, while the average response time of the browser-based experiment was 1,071 ms, yielding a difference of about 100 ms (Hilbig, 2016). However, it is important to note that within-participant variability was stable in the studies that reported reaction time delays, and therefore delayed reaction time did not affect the size of differences between conditions (Reimers and Stewart, 2015; de Leeuw and Motz, 2016; Hilbig, 2016; Semmelmann and Weigelt, 2017; Kochari, 2019).

One additional thing to note is that the participants in the present study were all undergraduate or graduate students from two colleges, and this population might be particularly motivated to pay close attention to the task, which might be why we obtained high-quality data. In the case of recruiting participants from heterogeneous groups using a crowdsourcing method, concerns have been raised that the participants will be easily distracted and the data quality may be poor (Woods et al., 2015). However, recent web-based crowdsourced experiments have successfully replicated standard lexical processing effects, as long as outliers are removed and other data-quality practices are adopted (Aguasvivas et al., 2020; Mandera et al., 2020). This all suggests that reliable data can be obtained using less-controlled web-based platforms, and further studies should be conducted using this approach.

Overall, the current study replicated the results reported in Lee et al. (2019) suggesting that print exposure measured by the KART can explain individual differences in online language processing using the lexical-decision task, as well as offline measures of language skills. In addition, the findings of the present study suggest that a web-based experimental procedure in which multiple behavioral measurements are collected can yield reliable data, indicating that these procedures are a valid alternative to well-controlled lab-based experiments, particularly as researchers are trying to find alternative ways to conduct studies in the global pandemic.

## Data Availability Statement

The raw data supporting the conclusions of this article will be made available by the authors, without undue reservation.

## Ethics Statement

The studies involving human participants were reviewed and approved by GIST Institutional Review Board. The patients/participants provided their written informed consent to participate in this study.

## Author Contributions

DK: conducting an experiment, analyzing data, and writing manuscript. ML: writing manuscript. WC: designing the experimental procedures, analyzing data, and writing manuscript. All authors contributed to the article and approved the submitted version.

## Conflict of Interest

The authors declare that the research was conducted in the absence of any commercial or financial relationships that could be construed as a potential conflict of interest.

## Publisher's Note

All claims expressed in this article are solely those of the authors and do not necessarily represent those of their affiliated organizations, or those of the publisher, the editors and the reviewers. Any product that may be evaluated in this article, or claim that may be made by its manufacturer, is not guaranteed or endorsed by the publisher.

## Footnotes

^1^Gender differences on LDT accuracy, reaction time, KART score, CRH score, and LS score were tested through independent-samples *t*-tests. Results revealed that there were no gender differences in LDT accuracy (*t* = −0.35, *df* = 139.81, *p* = 0.73), CRH (*t* = 1.62, *df* = 136.49, *p* = 0.11), or language skills tasks (*t* = −1.11, *df* = 129.53, *p* = 0.27). Meanwhile, LDT response time was marginally faster for men than women (*t* = −1.95, *df* = 125.7, *p* = 0.053), and KART scores were significantly higher in women than in men (*t* = −2.41, *df* = 133.97, *p* = 0.02). Differences on the measures between the two colleges were also examined through *t*-tests. Results showed that LDT accuracy, LDT response time, and KART scores were different between two colleges. Specifically, the LDT accuracy of GIST students was higher than that of Yeungnam University students (*t* = 2.50, *df* = 127.27, *p* = 0.01), and the LDT response time of GIST students was faster than Yeungnam University students (*t* = −2.35, *df* = 143.07, *p* = 0.02). Also, the KART score of GIST students was higher than that of Yeungnam University students (*t* = 5.61, *df* = 144.94, *p* < 0.001). On the other hand, there was no significant difference in CRH and language skills scores between the two colleges (CRH: *t* = 1.34, *df* = 143.06, *p* = 0.18; LS: *t* = 1.65, *df* = 144.22, *p* = 0.10).

^2^The same result was obtained when language ability score, a composite variable of KART, CRH, and LS (average *z* score of KART, CRH, and LS), was used as a fixed effect. Both the main effect of language ability scores (*ES* = −44.74, *SE* = 14.47, *t* = −10.49, *p* < 0.01) and the main effect of written word frequency (*ES* = −36.63, *SE* = 3.49, *t* = −3.09, *p* < 0.001) were observed, and the interaction between language ability scores and word frequency was also found (*ES* = 2.45, *SE* = 1.23, *t* = 2.00, *p* < 0.05). The pattern of the interaction was similar to the pattern observed for KART scores and word frequency. We also conducted a supplemental analysis in which gender and college were added as fixed effects to the original analysis. This analysis revealed main effects of word frequency (*ES* = −36.60, *SE* = 3.49, *t* = −10.49, *p* < 0.001) and college (*ES* = 71.66, SE = 20.85, *t* = 3.44, *p* < 0.001). Importantly, the interaction effect between word frequency and KART score also statistically significant (*ES* = 2.02, *SE* = 0.87, *t* = 2.32, *p* < 0.05). However, no main effects of KART scores (*ES* = −17.31, *SE* = 11.27, *t* = −1.54, *p* > 0.05) and gender (*ES* = 2.91, *SE* = 20.85, *t* = 0.15, *p* > 0.05) were found. This analysis shows that KART scores still modulate word frequency effects, even after controlling for the effects of gender and college.

^3^The same result was obtained when language ability score, a composite variable of KART, CRH, and LS (average *z* score of KART, CRH, and LS), was used as a fixed effect. Both the main effect of language ability scores (*ES* = 0.80, *SE* = 0.15, *z* = 5.19, *p* < 0.001) and the main effect of word frequency (*ES* = 0.54, *SE* = 0.066, *z* = 8.17, *p* < 0.001) were observed, and the interaction between language ability scores and word frequency was also found (*ES* = −0.067, *SE* = 0.028, *z* = −2.44, *p* < 0.05). The pattern of the interaction was similar to the pattern observed for KART scores and word frequency. Similar to the reaction time analysis, we also conducted an additional analysis adding gender and college as fixed effects to the original model of the RT analysis. This analysis revealed main effects of word frequency (*ES* = 0.55, *SE* = 0.07, *z* = 8.21, *p* < 0.001), KART score (*ES* = 0.67, *SE* = 0.13, *z* = 5.15, *p* < 0.001), gender (*ES* = −0.55, *SE* = 0.18, *z* = −3.03, *p* < 0.01), and college (*ES* = 0.54, *SE* = 0.19, *z* = 2.78, *p* < 0.01). The interaction effect between word frequency and KART scores also remained the same (*ES* = −0.06, *SE* = 0.02, *z* = −2.61, *p* < 0.01). This result indicates that the KART score still modulates word frequency effects, even after controlling for the effects of gender and college.
